# Adding exercise to usual care in patients with hypertension, type 2 diabetes mellitus and/or cardiovascular disease: a protocol for a systematic review with meta-analysis and trial sequential analysis

**DOI:** 10.1186/s13643-019-1233-z

**Published:** 2019-12-17

**Authors:** Anupa Rijal, Emil Eik Nielsen, Bianca Hemmingsen, Dinesh Neupane, Peter Haulund Gæde, Michael Hecht Olsen, Janus Christian Jakobsen

**Affiliations:** 1 Department of Internal Medicine, Holbæk Hospital, Holbæk, Denmark; 2 Department of Regional Health Research, The Faculty of Health Sciences, University of Southern Denmark, Odense, Denmark; 3 Department of Endocrinology and Nephrology, Nordsjællands Hospital, Hillerød, Denmark; 4 Welch Center for Prevention, Epidemiology, and Clinical Research, Department of Epidemiology, Johns Hopkins Bloomberg School of Public Health, Johns Hopkins University, Baltimore, USA; 5 Department of Cardiology and Endocrinology, Næstved-Slagelse-Ringsted (NSR) Hospital, Slagelse, Denmark; 6 Center for Individualized Medicine in Arterial Diseases, Odense University Hospital, University of Southern Denmark, Odense, Denmark; 7 Copenhagen Trial Unit, Rigshospitalet, Copenhagen, Denmark

**Keywords:** Hypertension, Type 2 diabetes mellitus, Cardiovascular disease, Exercise, Physical activity, Systematic review, Meta-analysis, Trial Sequential Analysis

## Abstract

**Background:**

Hypertension, type 2 diabetes mellitus and cardiovascular disease are among the leading causes of mortality globally. Exercise is one of the commonly recommended interventions/preventions for hypertension, type 2 diabetes mellitus and cardiovascular disease. However, the previous reviews have shown conflicting evidence on the effects of exercise. Our objective is to assess the beneficial and harmful effects of adding exercise to usual care for people with hypertension, type 2 diabetes mellitus and/or cardiovascular disease.

**Methods:**

This protocol for a systematic review was undertaken using the recommendations of The Cochrane Collaboration, the Preferred Reporting Items for Systematic Reviews and Meta-Analysis Protocols (PRISMA-P) and the eight-step assessment procedure suggested by Jakobsen et al. We plan to include all relevant randomised clinical trials and cluster-randomised trials assessing the effects of adding exercise to usual care for people with hypertension, type 2 diabetes mellitus and/or cardiovascular disease. We will search the Cochrane Central Register of Controlled Trials (CENTRAL), Medical Literature Analysis and Retrieval System Online (MEDLINE), Excerpta Medica database (EMBASE), Latin American and Caribbean Health Sciences Literature (LILACS), Science Citation Index Expanded on Web of Science, Chinese Biomedical Literature Database (CBM), China National Knowledge Infrastructure (CNKI), Chinese Science Journal Database (VIP) and BIOSIS. We will systematically assess the risks of random errors using Trial Sequential Analysis as well as risks of bias of all included trials. We will create a ‘Summary of Findings’ table in which we will present our primary and secondary outcomes, and we will assess the quality of evidence using the Grading of Recommendations Assessment, Development and Evaluation (GRADE).

**Discussion:**

The present systematic review will have the potential to aid patients, clinicians and decision-makers recommending exercise and thereby, benefit patients with hypertension, type 2 diabetes mellitus and/or cardiovascular disease.

**Systematic review registration:**

PROSPERO CRD42019142313

## Background

### Description of the conditions

Hypertension is the most important modifiable risk factor for cardiovascular disease globally [1, 2]. Hypertension is defined as a condition with an office systolic blood pressure equal to or above 140 mm Hg and/or diastolic blood pressure equal to or above 90 mm Hg [3]. Complications to hypertension account for 9.4 million deaths every year and contribute to 45% of deaths due to heart disease and 51% of deaths due to stroke [4]. From 2000 to 2010, the prevalence of hypertension has decreased by 2.6% in high-income countries while it has increased by 7.7% in low- and middle-income countries [5]. Despite the existence of effective blood pressure lowering drugs and preventive strategies, the burden of hypertension is constantly raising due to unhealthy lifestyle, ageing population [6] enhanced by lack of awareness and insufficient screening, treatment and control [7].

Cardiovascular disease is a common term for disorders of the heart or blood vessels which includes cerebrovascular disease, congenital heart disease, rheumatic heart disease, deep vein thrombosis, pulmonary thrombosis, coronary artery disease such as myocardial infarction, and heart failure [8]. It is the leading cause of death globally with 17.9 million deaths in 2016 [9]. Between 2007 and 2017, the age-standardised death rate due to cardiovascular disease decreased by about 10% globally [10]. However, it has escalated among low- and middle-income countries. Currently, more than three quarters of cardiovascular disease-related deaths occur in low- and middle-income countries [9]. The risk of cardiovascular disease is high among people with raised blood pressure (hypertension), raised blood glucose level and hyperlipidemia [8].

Patients with type 2 diabetes mellitus have defective insulin secretion and/or inadequate ability to utilise insulin produced by the body [11]. From 1980 to 2014, the prevalence of type 2 diabetes mellitus has doubled worldwide (4.7% vs 8.5%) [11]. The World Health Organization (WHO) estimates that by 2030, type 2 diabetes mellitus will be the 7th leading cause of death with predominant burden among urban dwellers from low- and middle-income countries [12, 13]. Long-term complications of type 2 diabetes mellitus lead to retinopathy, neuropathy and nephropathy, [11] and increase the risk of cardiovascular events by more than twofold [14] which heightens the risk of mortality and deteriorates the quality of life.

A substantial burden of cardiovascular disease in low- and middle-income countries may partly through hypertension, and type 2 diabetes mellitus be attributable to unhealthy lifestyle; physical inactivity, increased consumption of processed food, alcohol and tobacco abuse, high salt intake along with ageing population (decreasing infectious disease and famine) [15, 16]. Moreover, lack of adequate healthcare services which are often inaccessible and unaffordable for people living in this region create conducive environment for raising the burden of non-communicable diseases [17, 18]. Thus, it is important to ensure a comprehensive optimal lifestyle strategy to address the increasing burden of hypertension, type 2 diabetes mellitus and/or cardiovascular disease in low- and middle-income countries.

### Description of the intervention

Physical activity may be defined as ‘the movement of skeletal muscle that results in energy expenditure’ (Table 1) [19]. On the basis of major physiological effects, physical activity may be classified into aerobic, anaerobic, muscle strengthening activity, bone strengthening activity, flexibility, balance training activity and body mind therapies such as yoga or Tai Chi [20].

**Table 1:** Classification of physical activity based on MET

Metabolic Equivalent of Tasks (MET)*	Absolute intensity
1 to 1.5	Sedentary activity
1.6 to < 3.0	Light intensity activity
3.0 to < 6.0	Moderate-intensity activity
6 or higher	Vigorous intensity activity

^*^MET is a standard unit to measure physical activity. One MET is defined as 1 kcal/kg and is approximately equivalent to the energy cost of sitting quietly [20]

Exercise may be defined as ‘planned, structured and repetitive physical activities designed to improve or maintain physical fitness, physical performance and health’ [19]. Exercise is one of the most commonly recommended lifestyle interventions for people with hypertension, type 2 diabetes mellitus and/or cardiovascular disease [3, 19, 21]. Exercise occurs in various forms (Table 2). The intention with various forms of exercise in patients with hypertension, type 2 diabetes and/or cardiovascular disease is to enhance healthy structural, functional and biochemical characteristics eventually halting mortality [21–23]. A meta-epidemiological study suggested that structured exercise intervention was equally effective as drug intervention for prevention of mortality in the secondary prevention of coronary heart disease, rehabilitation after stroke, treatment of heart failure and prevention of diabetes [24]. Exercise seems to lower blood pressure, blood glucose, serum cholesterol and body weight, and seems to enhance insulin sensitivity, oxygen uptake and cardiac output [21, 23, 25, 26]. Physical inactivity is estimated as being the principal cause for approximately on third of both type 2 diabetes mellitus and ischaemic heart disease burden [27]. The recommended type and duration of exercise for patients with hypertension, type 2 diabetes mellitus and/or cardiovascular disease differ among regulatory bodies [3, 19, 21, 26, 28–31]. However, combination of aerobic and resistance exercises ranging from moderate to vigorous intensities has been recommended (Table 3). The optimal exercise for each patient may differ with individual progression of disease hence may need to be personalised [26].

**Table 2:** Common forms of exercise

Type of exercise	Description
Dynamic aerobic exercise	Various sustained exercises such as jogging, rowing, swimming or cycling that stimulate and strengthen the heart and lungs thereby improving the body’s utilisation of oxygen
Dynamic resistance exercise	Resistance exercise involves activities that use low or moderate repetition movements against resistance (dumbbells, bricks, rubber tubes, weight lifting) in an aim to increase strength, tone and endurance
Combined exercise	Combination of both aerobic exercise and resistance exercise
Isometric resistance exercise	Exercises that involve static contraction of muscles without joint movement such as planks, squat, shooting, horseback riding, judo and climbing
Body mind therapies	Activities such as yoga and Tai Chi which emphasises on relaxation, meditation which combines light aerobic activities, balancing and flexibility exercises

**Table 3:** Recommended level of exercise for patient with hypertension, type 2 diabetes mellitus and cardiovascular disease

Organisation	Recommended level of exercise for patient with hypertension, type 2 diabetes mellitus and cardiovascular disease
European Society of Cardiology	‘Hypertensive patient should participate in at least at least 30 min of moderate-intensity dynamic aerobic exercise (walking, jogging, cycling or swimming) on 5–7 days/week. Performance of resistance exercises on 2–3 days/week can also be advised [3, 28]’.
American Diabetes Association	‘Adults with diabetes should engage in 150 min or more of moderate-to-vigorous intensity activity weekly, spread over at least 3 days/week, with no more than 2 consecutive days without activity. Shorter durations (minimum 75 min/week) of vigorous intensity or interval training may be sufficient for younger and more physically fit individuals. In absence of contraindication, patient should be encouraged to perform resistance exercise at least twice per week [21]’.
American Heart Association	‘Endurance training of ≥ 5 day/week for 30–60 min and resistance training of 2–3 days/week for 30–45 min as therapy for cardiovascular patient [19]’.
Consensus for physical activity for Indian Asians	‘210 min per week of moderate-intensity physical activity for patient with cardiovascular disease and hypertension. Daily physical activity of 60-min duration including 10–15 min of resistance exercise and work-related activity should be maintained for all days of the week for patient with diabetes [29]’.

It should be noted that people in low- and middle-income countries may indulge in leisure time exercise, i.e. sports and exercise just as individuals from high-income countries [32]. However, their engagement in occupational and transportation or even household physical activity may surpass the recommended level of physical activity. Therefore, the importance of exercise for prevention of hypertension, type 2 diabetes mellitus and cardiovascular disease may be different in low- and middle-income countries.

### Why do we need this review?

We have identified several previous reviews of randomised clinical trials that have assessed the effects of exercise in patients with hypertension, type 2 diabetes mellitus and/or cardiovascular disease but with different conclusions (Table 4).

**Table 4:** Overview of existing reviews

First author	Title	Year of publication	Type of exercise	Information source	Primary outcome assessed	No. of trials	No. of patients	Published protocol	Assessment of adverse event	Assessment of risk of bias	Conclusion
Reviews assessing effectiveness of exercise in people with cardiovascular disease											
Powell [33]	Is exercise-based cardiac rehabilitation effective? A systematic review and meta-analysis to re-examine the evidence	2018	Exercise vs usual care (Type of exercise not specified)	Pubmed/Rehabilitation Medicine	Total mortality, cardiovascular mortality, hospital re-admission	22	4834	No	No	Yes (Risk of bias tool)	No reduction in total mortality and cardiovascular mortality
Anderson L [34]	Exercise based cardiac rehabilitation for coronary heart disease	2016	Exercise vs usual care (Type of exercise not specified)	Cochrane/Cochrane Database Syst Rev	Total mortality, cardiovascular mortality	63	14486	Yes	No	Yes (Risk of bias tool)	Reduction in total mortality but not cardiovascular mortality
Taylor RS [35]	Exercise-based rehabilitation for patients with coronary heart disease: systematic review and meta-analysis of randomized controlled trial	2004	Exercise vs usual care (Type of exercise not specified)	Pubmed/Am J Med	Total mortality, cardiovascular mortality	48	8940	No	No	Yes (Jadad Scale)	Reduction in both total mortality and cardiovascular mortality
Gloria Y. Yeh [36]	Tai Chi Exercise for Patients with Cardiovascular Conditions and Risk Factors: A Systematic Review	2010	Tai Chi	Pubmed/J Cardiopulm Rehabil Prev	Blood pressure, exercise capacity	14 (9 RCTs)	-	No	No	Yes (Quality Grading)	May have some benefit but inconclusive
Rees K [37]	Exercise based rehabilitation for heart failure (Review)	2004	Exercise vs usual care (Type of exercise not specified)	Cochrane/Cochrane Database Syst Rev	Total mortality, morbidity, hospital re-admission, physical capacity, quality of life	29	1126	Yes	Yes	Yes (Jadad Scale)	Exercise improved people’s fitness and quality of life, without causing harm but the trials included were small who are unrepresentative of the total population of patients with heart failure
Long L et al. [38]	Exercise-based cardiac rehabilitation for adults with stable angina	2018	Exercise vs usual care (Type of exercise not specified)	Cochrane/Cochrane Database Syst Rev	All-cause mortality, morbidity health-related quality of life (e.g. (SF-36), (EQ-5D), exercise capacity (e.g VO2peak, 6-min walk test), cardiovascular-related hospital admissions	8	581	Yes	Yes	Yes (Risk of bias tool)	Small trials, potential risk of bias and concerns about imprecision and lack of applicability, the effects of exercise-based CR compared with control on mortality, morbidity, cardiovascular hospital admissions, adverse events, return to work and health-related quality of life in people with stable angina was uncertain
Saunders DL et al. [39]	Physical fitness training for stroke patients	2016	Cardiorespiratory training, resistance training, mixed training	Cochrane/Cochrane Database Syst Rev	Case fatality, death or dependence (Barthel Index score, Rankin score), disability (e.g. Functional Independence Measure, Stroke Impact scale etc.)	45	2188	Yes	Yes	Yes (Risk of bias tool)	Cardiorespiratory fitness training can improve exercise ability and walking after stroke. Further well-designed randomised trials are needed to determine the optimal exercise prescription and identify long-term benefits
Ismail et al. [40]	Clinical Outcomes and Cardiovascular Responses to Different Exercise Training Intensities in Patients With Heart Failure A Systematic Review and Meta-Analysis	2013	Aerobic exercise training	JACC/Heart Fail	Peak VO2 (baseline and after exercise), training frequency, intensity, duration per session, length of program, participant completion rates, mortality, adverse medical events and hospitalisations	74	5877	No	Yes	Yes (PEDrO Scale)	Magnitude of gain in cardiorespiratory fitness is greater with increasing exercise intensity. High and vigorous exercise intensities did not appear to increase the risk for study withdrawal, death, adverse events and hospitalisation
Davies EJ et al. [41]	Exercise training for systolic heart failure: Cochrane systematic review and meta-analysis	2010	Exercise versus usual care (Type of exercise not specified)	Pubmed/European Journal of Heart Failure	All-cause mortality, hospital admission/re-admission rates, HRQoL assessed by a validated outcome measure (e.g. MLWHF questionnaire or Short Form 36 (SF-36)) and cost-effectiveness	19	3647	No	No	Yes (Risk of bias tool)	No significant difference between exercise and control in short-term (≤ 12 months) or longer term all-cause mortality or overall hospital admissions.
Reviews assessing effectiveness of exercise in people with hypertension											
Whelton SP et al. [42]	Effect of Aerobic Exercise on Blood Pressure: A Meta-Analysis of Randomized, Controlled Trials	2002	Aerobic exercise	Pubmed/Annals of Internal Medicine	Blood pressure	54	2419	No	No	No	Aerobic exercise reduces blood pressure in both hypertensive and normotensive persons.
MacDonald HV et al. [43]	Dynamic Resistance Training as Stand-Alone Antihypertensive Lifestyle Therapy: A Meta-Analysis	2016	Dynamic resistance training	Pubmed/J Am Heart Assoc	Blood pressure	64	2344	No	No	No	For non-white adult samples with hypertension, dynamic RT may elicit BP reductions that are comparable with or greater than those reportedly achieved with AE training
Cornelissen et al. [44]	Exercise Training for Blood Pressure: A Systematic Review and Meta-analysis	2013	Endurance, Resistance, Isometric resistance, Combined exercise	Pubmed/J Am Heart Assoc.	Blood pressure	93	5223	No	No	No	Endurance, dynamic resistance and isometric resistance training lower SBP and DBP, whereas combined training lowers only DBP.
Cramer H et al. [45]	A Systematic Review and Meta-Analysis of Yoga for Hypertension	2014	Yoga	Pubmed/Am J Hypertens	Blood pressure	7	452	No	No	Yes (Risk of bias tool)	Larger studies are required to confirm the emerging but low-quality evidence that yoga may be a useful adjunct intervention in the management of hypertension
Chu P et al. [46]	The effectiveness of yoga in modifying risk factors for cardiovascular disease and metabolic syndrome: A systematic review and meta-analysis of randomized controlled trials	2016	Yoga	Pubmed/Eur J Prev Cardiol	BMI, systolic blood pressure, low-density lipoprotein cholesterol, high-density lipoprotein cholesterol	37	-	No	No	Yes (Risk of bias tool)	Promising evidence of yoga on improving cardio-metabolic health. Findings are limited by small trial sample sizes, heterogeneity and moderate quality of RCTs.
Hagins M et al. [47]	Effectiveness of Yoga for Hypertension: Systematic Review and Meta-Analysis	2013	Yoga	Pubmed/Evid Based Complement Alternat Med	Systolic and diastolic blood pressure	17	-	No	No	Yes (Risk of bias tool)	Yoga can be preliminarily recommended as an effective intervention for reducing blood pressure. Additional rigorous controlled trials are warranted to further investigate the potential benefits of yoga.
Reviews assessing effectiveness of exercise in type 2 diabetes mellitus											
Thomas D [48]	Exercise for type 2 diabetes mellitus (Review)	2009	Aerobic, fitness or progressive resistance training exercise	Cochrane/Cochrane Database Syst Rev	HbA1c	14	377	Yes	Yes	Yes (Risk of bias tool)	Reduced HbA1c even without reducing weight. No trials included reported mortality. No adverse event was reported.
Hayashino Y [49]	Effects of supervised exercise on lipid profiles and blood pressure control in people with type 2 diabetes mellitus: A meta-analysis of randomized controlled trials	2012	Aerobic, resistance or combined	Pubmed/Diabetes Research and Clinical Practice	Blood pressure and lipid profile	42	-	No	No	Yes (Verhagen et. al's tool)	Supervised exercise is effective in improving blood pressure and lipid profile.
Grace A et al. [50]	Clinical outcomes and glycaemic responses to different aerobic exercise training intensities in type 2 diabetes: a systematic review and meta-analysis	2017	Aerobic exercise	Pubmed	% change in HbA1c	27	1372	No	No	Yes (TESTEX)	Improvement in HbA1c. Higher intensity of exercise gives bigger benefit.
Snowling NJ et al. [51]	Effects of Different Modes of Exercise Training on Glucose Control and Risk Factors for Complications in Type 2 Diabetic Patients. A meta-analysis	2006	Aerobic Resistance Combined	Pubmed/Diabetes care	Glucose control HbA1c	27	-	No	No	No	All forms of exercise training produce small benefits in the main measure of glucose control: HbA1c
Liu Y et al. [52]	Resistance Exercise Intensity is Correlated with Attenuation of HbA1c and Insulin in Patients with Type 2 Diabetes: A Systematic Review and Meta-Analysis	2019	Resistance	Pubmed/Int J Environ Res Public Health	HbA1c Insulin	24	962	No	No	Yes (Risk of bias tool)	High-intensity RE has greater beneficial effects than low-to-moderate-intensity in attenuation of HbA1c and insulin in T2D patients.
Schwingshackl L et al. [25]	Impact of different training modalities on glycaemic control and blood lipids in patients with type 2 diabetes: a systematic review and network meta-analysis	2014	Aerobic, Resistance, Combined	Pubmed/Diabetologia	HbA1c	14	915	No	No	Yes (Risk of bias tool)	Combined interventions resulted in significantly more pronounced improvements in glycaemic control
Innes KE et al. [53]	Yoga for Adults with Type 2 Diabetes: A Systematic Review of Controlled Trials	2015	Yoga	Pubmed/Journal of Diabetes Research	Glycaemia and insulin resistance, lipid profile, body weight and composition, blood pressure	33 (12 RCTs)	.	No	No	Yes (PEDrO Scale)	Methodological limitation of existing evidence to report beneficial effect of yoga
Ciu J et al. [54]	Effects of yoga in adults with type 2 diabetes mellitus: A meta-analysis	2017	Yoga	Pubmed/Journal of Diabetes Investigation	Fasting blood glucose	12	864	No	No	Yes (Jadad Scale)	Methodological limitation and possible heterogeneity cannot confirm the beneficence of yoga, further studies needed.
Chao et al. [55]	The Effects of Tai Chi on Type 2 Diabetes Mellitus: A Meta-Analysis	2018	Tai Chi	Pubmed/Journal of Diabetes Research	Fasting blood glucose	14	798	No	No	Yes (Jadad Scale)	Tai chi can effectively affect the management of blood glucose and HbA1c in type-2 DM patients
Xia TW et al. [56]	Different training durations and styles of tai chi for glucose control in patients with type 2 diabetes: a systematic review and meta-analysis of controlled trials	2019	Tai Chi	Pubmed/BMC Complementary and Alternative Medicine	HbA1c and fasting blood glucose	17	-	No	2 trials reported no adverse event Rest did not report	Yes (Risk of bias tool)	Tai Chi seems to be effective in treating type 2 diabetes. Different training durations and styles result in variable effectiveness
Lee MS et al. [57]	Tai Chi for Management of Type 2 Diabetes Mellitus: A Systematic Review	2011	Tai Chi	Pubmed/Chin J Integr Meed	HbA1c and fasting blood glucose, quality of life	10	-	No	No	No	Exiting evidence does not suggest Tai chi is effective. There are few high-quality trials on which to make definitive judgements.

A review published in 2004 reported that exercise-based cardiac rehabilitation for coronary heart patients reduced all-cause mortality and cardiac mortality irrespective of type of cardiac rehabilitation (exercise only or in combination of psychosocial/educational intervention) or duration of the exercise [35]. Another Cochrane review published in 2016 on exercise-based cardiac rehabilitation exercise compared with no exercise reported that the exercise-based cardiac rehabilitation reduced the risk of cardiovascular mortality but not the risk of all-cause mortality [34], while the most recent update of the review in 2018 reported that exercise-based cardiac rehabilitation did not influence total mortality nor cardiac mortality [33]. Similarly, another meta-analysis reported that there was no significant difference between exercise (short term; ≤ 12 months or long term) compared with usual care on all-cause mortality or overall hospital admissions among patient with heart failure [41]. Another Cochrane review on the effectiveness of exercise-based rehabilitation after heart failure [37] and stable angina [38] reported uncertainty of the effects of exercise on prevention of mortality due to lack of sufficient randomised clinical trials with longer follow-up reporting mortality. Likewise, a Cochrane review on physical training after stroke [39] reported improvement in movement, exercise capacity and quality of life. However, the trials in the review did not report on mortality. Another meta-analysis among patients with heart failure showed no significant difference in mortality between different intensities of exercise [40].

The blood pressure lowering effect of various kinds of exercise has been reported by previous meta-analysis [42–44]. A recent meta-analysis reported that exercise interventions appear to be as equally effective as most antihypertensive medications on reducing systolic blood pressure [58] in patients with hypertension. However, across the whole population, the reduction in systolic blood pressure was greater among those receiving antihypertensive medication as compared with individuals who adopted structured exercise regimens [58].

Likewise, previous meta-analysis of randomised clinical trials on effect of different kinds of exercises among people with type 2 diabetes mellitus has recurrently reported reductions of blood glucose levels (glycated haemoglobin HbA1c) [25, 48, 50–52], while another meta-analysis showed supervised exercise among people with type 2 diabetes mellitus leads to reduction of systolic and diastolic blood pressure and lipid profiles irrespective of type of exercise involved [49].

A systematic review on Tai Chi exercise as an intervention for patient with cardiovascular disease was inconclusive on effectiveness of the therapy due to lack of high-quality randomised clinical trials [36]. Similar insufficiency of evidence on impact of yoga [45–47, 54] and Tai Chi [53, 57] among people with hypertension and type 2 diabetes mellitus exists. Two recent meta-analyses showed that Tai Chi is effective in reducing fasting blood glucose and glycated haemogloin (HbA1c) [55, 56] among patients with type 2 diabetes mellitus. However, majority of the trials included in both the reviews were of poor methodological quality and showed publication bias.

We have not identified any systematic review considering both risks of systematic errors and random errors assessing the effects of adding exercise to usual care in patients with hypertension, type 2 diabetes mellitus and/or cardiovascular disease for prevention of mortality. Furthermore, the existing reviews are outdated. No prior systematic review has attempted to assess the effect of adding exercise in usual care especially for low- and middle-income countries for prevention of mortality. Thus, we also aim to conduct a subgroup analysis for assessing effect of adding exercise to usual care on prevention of mortality for low- and middle-income countries.

## Methods

### Objective

To assess the beneficial and harmful effects of adding exercise to usual care for people with hypertension, type 2 diabetes mellitus and/or cardiovascular disease.

### Methods

This protocol for a systematic review has been developed based on the Preferred Reporting Items for Systematic Reviews and Meta-Analysis Protocols (PRISMA-P) guidelines for reporting systematic reviews evaluating interventions in healthcare [59, 60] (see Additional file 1).

### Criteria for considering studies for this review

#### Types of studies

Randomised clinical trials and cluster-randomised trials irrespective setting, trial duration, publication status, publication year and language. We will not include quasi-randomised studies or observational studies.

#### Types of participants

We will include adult participants (as defined by trialists) with hypertension (as defined by trialists), type 2 diabetes mellitus (as defined by trialists) and/or any cardiovascular disease (as defined by the trialists) or cardiovascular disease as defined by WHO. It includes cerebrovascular disease, congenital heart disease, rheumatic heart disease, deep vein thrombosis, pulmonary thrombosis, coronary artery disease such as myocardial infarction, and heart failure [8].

#### Types of interventions

- Experimental intervention: exercise (or similar terms used by the trialists) see Table 2.
- Control intervention: no exercise (primary comparison) or usual care (or similar terms defined by the trialists).
- Co-interventions: any co-intervention, if the co-intervention is intended to be delivered similarly to the intervention and control groups.

### Outcomes

#### Primary outcomes

1. All-cause mortality.
2. Proportion of participants with a serious adverse event defined as any untoward medical occurrence that resulted in death; was life threatening; was persistent; or led to significant disability, nephrotoxicity, superinfection, need for respiratory support, need for circulatory support or prolonged hospitalisation [61]. As we expect the trialists’ reporting of serious adverse events to be heterogeneous and not strictly according to the International Council for Harmonisation guideline for Good Clinical Practice (ICH-GCP) recommendations, we will include the event as a serious adverse if the trialists either (1) use the term ‘serious adverse event’ but not refer to ICH-GCP or (2) report the proportion of participants with an event we consider fulfils the ICH-GCP definition (e.g. myocardial infarction or hospitalisation). If several of such events are reported, then we will choose the highest proportion reported in each trial.
3. Quality of life measured on any valid scale (e.g. Short Form-36).

#### Secondary outcomes

1. Cardiovascular mortality (as defined by trialists).
2. Blood pressure (both systolic and diastolic).
3. Microvascular complications (retinopathy, nephropathy, neuropathy, diabetic foot).
4. Myocardial infarction (as defined by trialists).
5. Stroke (as defined by trialists).

#### Exploratory outcomes

1. Body weight (kg).
2. Exercise capacity assessed by validated outcome measure (e.g.VO_2 max,_ 6-min walk test).

For all outcomes, we will use the trial results reported to the longest follow-up.

### Search methods for identification of studies

#### Electronic searches

We will search the Cochrane Central Register of Controlled Trials (CENTRAL), Medical Literature Analysis and Retrieval System Online (MEDLINE), Excerpta Medica database (EMBASE), Latin American and Caribbean Health Sciences Literature (LILACS), Science Citation Index Expanded on Web of Science, Chinese Biomedical Literature Database (CBM), China National Knowledge Infrastructure (CNKI), Chinese Science Journal Database (VIP) and BIOSIS to identify relevant trials. The preliminary search strategy for MEDLINE (Ovid) is given in Appendix. We will search all databases from their inception to the present.

#### Searching other resources

The reference lists of relevant publications will be checked for any unidentified randomised trials.

Further, we will search for ongoing trials on the following:

- _ ClinicalTrials.gov (www.clinicaltrials.gov)
- _ Google Scholar (https://scholar.google.com/)
- _ The Turning Research into Practice (TRIP) Database
- (https://www.tripdatabase.com)
- _ Medicines and Healthcare products Regulatory
- Agency ( https://www.gov.uk/government/organisations/medicines-and-healthcare-products-regulatory-agency)
- _ The World Health Organization (WHO) International Clinical Trials Registry Platform (ICTRP) search portal (http://apps.who.int/trialsearch)

Additionally, we will by hand search conference abstracts from cardiology and diabetes conferences for relevant trials. We will consider unpublished and grey literature trials relevant to the review, if we identify such trials.

### Data collection and analysis

We will perform the review following the recommendations of The Cochrane Collaboration [62]. The analyses will be performed using STATA 16 and Trial Sequential Analysis [63].

### Selection of studies

Two review authors (AR and EEN) will independently screen titles and abstracts. We will retrieve all relevant full-text study reports and publications. Two review authors (AR and EEN) will independently screen the full text and identify and record reasons for exclusion of the ineligible studies. We will resolve any disagreement through discussion or, if required, we will consult a third author (JCJ). Trial selection will be displayed in an adapted flow diagram as per the Preferred Reporting Items for Systematic Reviews and Meta-Analyses (PRISMA) statement [59].

### Data extraction and management

Two authors (AR and EEN) will extract data independently from included trials. Disagreements will be resolved by discussion with a third author (JCJ). We will assess duplicate publications and companion papers of a trial together to evaluate all available data simultaneously (maximise data extraction, correct bias assessment). We will contact the trial authors by email to specify any additional data, which may not have been reported sufficiently or at all in the publication.

### Trial characteristics

Bias risk components (as defined below), trial design (parallel, cluster, factorial or crossover), number of intervention arms, length of follow-up, estimation of sample size and inclusion and exclusion criteria.

### Participant characteristics and diagnosis

Number of participants with cardiovascular disease (as defined by trialists); number of participants with type 2 diabetes mellitus (as defined by trialists); number of randomised participants; number of analysed participants; number of participants lost to follow-up/withdrawals/crossover; age range (mean or median) and sex ratio. We will additionally report the proportion of participants in the compared groups who receive exercise (or similar term used by trialists).

### Exercise characteristics

Exercise characteristics are as follows: type of exercise, duration of exercise, frequency of exercise and follow-up period.

### Co-intervention characteristics

Co-intervention characteristics are as follows: type of co-intervention, duration of co-intervention and frequency of co-intervention.

### Individual patient data

We will ask those responsible for the included trials to supply individual patient data. *Notes*: Funding of the trial and notable conflicts of interest of trial authors will be extracted, if available. We will note in the ‘Characteristics of included studies’ table if outcome data were not reported in a usable way. Two review authors (AR and EEN) will independently transfer data into the Review Manager file. Disagreements will be resolved through discussion, or if required, we will consult with a third author (JCJ).

### Assessment of risk of bias in included studies

We will use the instructions given in the *Cochrane Handbook for Systematic Reviews of Interventions* in our evaluation of the methodology and hence the risk of bias of the included trials.

We will classify the trials according to the following criteria.

#### Random sequence generation

- Low risk: If sequence generation was achieved using computer random number generator or a random number table. Drawing lots, tossing a coin, shuffling cards and throwing dice were also considered adequate if performed by an independent adjudicator.
- Unclear risk: If the method of randomisation was not specified, but the trial was still presented as being randomised.
- High risk: If the allocation sequence is not randomised or only quasi-randomised. These trials will be excluded.

#### Allocation concealment

- Low risk: If the allocation of patients was performed by a central independent unit, on-site locked computer, identical-looking numbered sealed envelopes, drug bottles or containers prepared by an independent pharmacist or investigator.
- Uncertain risk: If the trial was classified as randomised but the allocation concealment process was not described.
- High risk: If the allocation sequence was familiar to the investigators who assigned participants.

#### Blinding of participants and treatment providers

- Low risk: If the participants and the treatment providers were blinded to intervention allocation and this was described.
- Uncertain risk: If the procedure of blinding was insufficiently described.
- High risk: If blinding of participants and the treatment providers was not performed.

#### Blinding of outcome assessment

- Low risk of bias: If it was mentioned that outcome assessors were blinded and this was described.
- Uncertain risk of bias: If it was not mentioned if the outcome assessors in the trial were blinded or the extent of blinding was insufficiently described.
- High risk of bias: If no blinding or incomplete blinding of outcome assessors was performed.

#### Incomplete outcome data

- Low risk of bias: If missing data were unlikely to make treatment effects depart from plausible values. This could be either (1) there were no drop-outs or withdrawals for all outcomes or (2) the numbers and reasons for the withdrawals and drop-outs for all outcomes were clearly stated and could be similar to both groups. Generally, the trial is judged as at a low risk of bias due to incomplete outcome data if drop-outs are less than 5%. However, the 5% cut-off is not definitive.
- Uncertain risk of bias: If there was insufficient information to assess whether missing data were likely to induce bias on the results.
- High risk of bias: If the results were likely to be biased due to missing data either because the pattern of drop-outs could be different in the two intervention groups or the trial used improper methods in dealing with the missing data (e.g. last observation carried forward).

#### Selective outcome reporting

- Low risk of bias: If a protocol was published before or at the time the trial began and the outcomes specified in the protocol were reported. If there was no protocol or the protocol was published after the trial has begun, reporting of all-cause mortality and serious adverse events will grant the trial a grade of low risk of bias.
- Uncertain risk of bias: If no protocol was published and the outcome all-cause mortality and serious adverse events were not reported on.
- High risk of bias: If the outcomes in the protocol were not reported on.

#### For profit bias

- Low risk of bias: If the trial appeared to be free of other components of for-profit bias.
- Unclear risk of bias: If it was unclear whether the trial was free of for-profit bias.
- High risk of bias: If there was a high risk of for-profit bias.

#### Other risks of bias

- Low risk of bias: If the trial appears to be free of other components (for example, academic bias or for-profit bias) that could put it at risk of bias.
- Unclear risk of bias: If the trial may or may not be free of other components that could put it at risk of bias.
- High risk of bias: If there are other factors in the trial that could put it at risk of bias (for example, authors conducted trials on the same topic, for- profit bias, etc.).

#### Overall risk of bias

- Low risk of bias: The trial will be classified as overall ‘low risk of bias’ only if all of the bias domains described in the above paragraphs are classified as ‘low risk of bias’.
- High risk of bias: The trial will be classified as ‘high risk of bias’ if any of the bias risk domains described in the above are classified as ‘unclear’ or ‘high risk of bias’.

These components enable classification of randomised trials with low risk of bias and high risk of bias. The latter trials tend to overestimate positive intervention effects and underestimate negative effects [64–70]. We will classify a trial as being at overall ‘low risk of bias’, only if all bias domains are classified as ‘low risk of bias’. We will classify a trial as being at overall ‘high risk of bias’, if any of the bias domains are classified as ‘unclear’ or ‘high risk of bias’.

We will assess the domains ‘blinding of outcome assessment’, ‘incomplete outcome data’ and ‘selective outcome reporting’ for each outcome result. Thus, we can assess the bias risk for each outcome assessed in addition to each trial. Our primary conclusions will be based on the results of our primary outcomes at overall low risk of bias.

### Differences between the protocol and the review

We will conduct the review according to this published protocol and report any deviations from it in the ‘Differences between the protocol and the review’ section of the systematic review.

### Measures of treatment effect

#### Dichotomous outcomes

We will calculate risk ratios (RRs) with 95% confidence interval (CI) for dichotomous outcomes, as well as the Trial Sequential Analysis-adjusted CIs (see below).

#### Continuous outcomes

We will calculate the mean differences (MDs) with 95% CI for continuous outcomes as well as the Trial Sequential Analysis-adjusted CIs (see below). We will consider assessing health-related quality of life across different scales with standardised mean difference [62].

### Dealing with missing data

We will, as first option, contact all trial authors to obtain any relevant missing data (i.e. for data extraction and for assessment of risk of bias, as specified above).

#### Dichotomous outcomes

We will not impute missing values for any outcomes in our primary analysis. In two of our sensitivity analyses (see paragraph below), we will impute data.

#### Continuous outcomes

We will primarily analyse scores assessed at single time points. If only changes from baseline scores are reported, we will analyse the results together with follow-up scores. If standard deviations (SDs) are not reported, we will calculate the SDs using trial data, if possible. We will not use intention-to-treat data if the original report did not contain such data. We will not impute missing values for any outcomes in our primary analysis. In our sensitivity analysis (see paragraph below) for continuous outcomes, we will impute data.

### Assessment of heterogeneity

We will primarily investigate forest plots to visually assess heterogeneity. We will secondly assess the presence of statistical heterogeneity by chi-square test (threshold *P* < 0.10) and measure the quantities of heterogeneity by the *I*^2^ statistics [71, 72].

We will investigate possible heterogeneity through subgroup analyses. Ultimately, we may decide that a meta-analysis should be avoided [62].

### Assessment of reporting biases

We will use a funnel plot to assess reporting bias if ten or more trials are included. We will visually inspect funnel plots to assess the risk of bias. We are aware of the limitations of a funnel plot (i.e. a funnel plot assesses bias due to small sample size). From this information, we assess possible reporting bias. For dichotomous outcomes, we will test asymmetry with the Harbord test [73] if τ2 is less than 0.1 and with the Rücker test if τ2 is greater than 0.1. For continuous outcomes, we will use the regression asymmetry test [74] and the adjusted rank correlation test [75].

### Unit of analysis issues

We will only include randomised clinical trials and cluster-randomised trials. For trials using crossover design, only data from the first period will be included [62, 76]. We will include cluster-randomised trials after adjusting the original sample size of the trial to the effective sample size using the intracluster correlation coefficient from the ‘design effect’ [62]. Therefore, there will not be any unit of analysis issues.

### Data synthesis

#### Meta-analysis

We will undertake this meta-analysis according to the recommendations stated in the Cochrane Handbook for Systematic Reviews of Interventions [62], Keus et al. [77] and the eight-step assessment suggested by Jakobsen et al. [78]. We will use the statistical software Review Manager 5.3 provided by Cochrane to analyse data [79].

We will assess our intervention effects with both random effects meta-analyses [80] and fixed effects meta-analyses [81]. We will use the more conservative point estimate of the two [78]. The more conservative point estimate is the estimate closest to zero effect. If the two estimates are similar, we will use the estimate with the highest *P* value.

We use three primary outcomes, and therefore, we will consider a *P* value of 0.025 as the threshold for statistical significance [78]. For secondary and exploratory outcomes, we will consider a *P* value of 0.05 as the threshold for statistical significance as we only consider these results as hypothesis generating. We will investigate possible heterogeneity through subgroup analyses. Ultimately, we may decide that a meta-analysis should be avoided because of unexpected high heterogeneity [62]. We will use the eight-step procedure to assess if the thresholds for significance are crossed [78]. Our primary conclusion will be based on the results from the primary outcomes at low risk of bias [78]. Where multiple trial groups are reported in a single trial, we will include only the relevant groups. If two comparisons are combined in the same meta-analysis, we will have the control group to avoid double-counting [62]. Trials with a factorial design will be included.

#### Trial Sequential Analysis

Traditional meta-analysis runs the risk of random errors due to sparse data and repetitive testing of accumulating data when updating reviews. We wish to control the risks of type I errors and type 2 errors. We will, therefore, perform Trial Sequential Analysis on the outcomes, in order to calculate the required information size (that is the number of participants needed in a meta-analysis to detect or reject a certain intervention effect) and the cumulative *Z*-curve’s breach of relevant trial sequential monitoring boundaries [63, 82–90]. A more detailed description of Trial Sequential Analysis can be found in the Trial Sequential Analysis manual and at http://www.ctu.dk/tsa/ [88]. For dichotomous outcomes, we will estimate the required information size based on the observed proportion of patients with an outcome in the control group (the cumulative proportion of patients with an event in the control groups relative to all patients in the control groups), a relative risk reduction of 25%, an alpha of 2.5% for primary outcomes, an alpha of 5% for secondary and exploratory outcomes, a beta of 10%, and diversity as suggested by the trials in the meta-analysis. For continuous outcomes, we will in the Trial Sequential Analysis use the observed SD, a mean difference of the observed SD/2, an alpha of 2.5% for primary outcomes, an alpha of 5% for secondary and exploratory outcomes, and a beta of 10%.

### Subgroup analysis and investigation of heterogeneity

#### Subgroup analysis

We will perform the following subgroup analysis when analysing the primary outcomes (all-cause mortality, serious adverse event and quality of life):

1. Trials at high risk of bias compared with trials at low risk of bias
2. Different types of exercise (Table 2)
3. High-income countries compared with low- and middle-income countries
4. Men compared with women
5. Different disease group: hypertension, type 2 diabetes mellitus and/or cardiovascular disease

We will use the formal test for subgroup interactions in Review Manager [91].

#### Sensitivity analysis

To assess the potential impact of the missing data for dichotomous outcomes, we will perform the two following sensitivity analyses on both the primary and secondary outcomes:

‘Best-worst-case’ scenario: we will assume that all participants lost to follow-up in the exercise group have survived, had no serious adverse events and had a higher quality of life (see paragraph below). We will assume the opposite for all participants lost to follow-up in the control group.

‘Worst-base-case’ scenario: we will assume that all participants lost to follow-up in the exercise have not survived, had serious adverse events and had a lower quality of life (see paragraph below). We will assume the opposite for all participants lost to follow-up in the control group. We will present results of both scenarios in our review. When analysing quality of a ‘beneficial outcome’ will be the group mean plus two standard deviations (SDs) of the group mean and a ‘harmful outcome’ will be the group mean minus two SDs of the group mean [78]. We will present results of this scenario in our review. Other post hoc sensitivity analyses might be warranted if unexpected clinical or statistical heterogeneity is identified during the analysis of the review results [78].

### ‘Summary of Findings’ table

We will create a ‘Summary of Findings’ table using each of the pre-specified outcomes (all-cause mortality, serious adverse event, quality of life, cardiovascular death, blood pressure, microvascular complications and myocardial infarction, stroke). We will use the five GRADE considerations (bias risk of the trials, consistency of effect, imprecision, indirectness and publication bias) to assess the quality of a body of evidence as it relates to the studies which contribute data to the meta-analyses for the pre-specified outcomes [78, 92–94]. We will assess ‘imprecision’ using Trial Sequential Analysis; otherwise, we will use methods and recommendations described in Chapter 8 (Section 8.5) and Chapter 12 of the *Cochrane Handbook for Systematic Reviews of Interventions* [62].

## Discussion

Our planned methodology has several strengths. We have based the protocol on the Preferred Reporting Items for Systematic Reviews and Meta-Analysis Protocols (PRISMA-P) checklist [59, 60]. We have pre-defined our methodology based on *the Cochrane Handbook for Systematic Reviews of Interventions* [62], Keus et al. [77], the eight-step assessment as suggested by Jakobsen et al. [78], Trial Sequential Analysis [63] and the GRADE assessment [94, 95]. Through our pre-defined methodology, we consider both risks of random errors and systematic errors.

Our planned methodology also has limitations. We will pool data from all trials regarding adding different types of exercise to usual care for individuals with hypertension, type 2 diabetes mellitus and/or cardiovascular disease, which potentially will lead to clinical and statistical heterogeneity. To take this potential heterogeneity into account, we have pre-defined several subgroup and sensitivity analysis to assess whether or not the intervention effects differ between conditions and trials, and we will ultimately decide if meta-analysis should be avoided. Another limitation is that we do not search for non-randomised studies, and hence we may overlook harms [96]. If the present review finds solid evidence for benefits, then a more thorough investigation of potential harms seems warranted.

With this systematic review, we seek to provide the clinicians and decision-makers with a reliable evidence adjusted for bias, sparse data, and multiple testing regarding the beneficial and harmful effects of adding exercise to usual care for people with hypertension, type 2 diabetes mellitus and/or cardiovascular disease.

### Supplementary information

**Additional file 1:** PRISMA-P 2015 Checklist for this protocol.

## Data Availability

Not applicable

## Appendix

### Preliminary search strategy Ovid _Medline (1946 to July 2019)

#### Cardiovascular diseases

1. exp Cardiovascular Diseases/
2. ((heart or cardiac or cardial or cardiopath* or cardiomyopath* or coronor* or myocord*) adj3 (ischem* or ischaem* or anoxia or hypoxia)).ab,ti
3. (coronary adj3 (insufficien* or occlus* or disease* or acute or atherosclero* or orteriosclero* or sclero* or cordiosclero* or constrict* or vasoconstrict* or obstruct* or stenosis* or thrombo*)).ab,ti
4. ((heart or myocard* or cardiac or cardial) adj3 infarct*).ab,ti
5. ((cerebrovascul* or brain or 'cerebral vascular' or cerebrovascular) adj3 (accident* or lesion or attack or ischem* or ischaem* or insult* or insuffucien* or arrest* or apoplex*)).ab,ti
6. (cva or stroke or angina*) .ab,ti
7. exp Hypertension/
8. (pressure adj3 (blood or systolic or diastolic or arterial or venous or pulse)).ab,ti
9. (hypertensive or antihypertensive or anti-hypertensive).ab,ti
10. 1 or 2 or 3 or 4 or 5 or 6 or 7 or 8 or 9

#### Type 2 diabetes mellitus

11. exp Diabetes Mellitus, Type 2/
12. (('adult onset' or 'type 2' or 'type II' or 'non-insulin dependent' or 'noninsulin dependent' or 'insulin independent') adj3 diabet*).ab,ti
13. 11 or 12

#### Exercise

14. exp Exercise/ or exp Exercise Therapy/
15. exp Sports/
16. exp Physical Exertion/
17. rehabilitat*.ab,ti
18. (physical* adj3 (fit* or train* or therap* or activit*)).ab,ti
19. (train* adj3 (strength* or aerobic* or exercise* or endurance* or weight*)).ab,ti
20. ((exercise* or fitness) adj3 (treatment or intervent* or program* or rehabilitat*)).ab,ti
21. ((exercise* adj3 (aerobic* or endurance* or combine* or resistance*)).ab,ti
22. (run* or bicycle* or treadmill* or ergometer* or walk* or swim*).ab,ti
23. exp Tai Ji/
24. (‘tai chi’ or ‘tai chi chuan’ or ‘ta'i chi’ or ‘tai ji’ or taijiquan).ab,ti
25. exp Yoga/th [Therapy]
26. 14 or 15 or 16 or 17 or 18 or 19 or 20 or 21 or 22 or 23 or 24 or 25
27. **10 or 13 and 26**
28. random*.mp. [mp=title, abstract, original title, name of substance word, subject heading word, floating sub-heading heading word, keyword heading word, protocol supplementary concept word, rare disease supplementary concept word, unique identifier, synonyms]

29. blind*.mp. [mp=title, abstract, original title, name of substance word, subject heading word, floating sub-heading heading word, keyword heading word, protocol supplementary concept word, rare disease supplementary concept word, unique identifier, synonyms]

30. meta-analys*.mp. [mp=title, abstract, original title, name of substance word, subject heading word, floating sub-heading heading word, keyword heading word, protocol supplementary concept word, rare disease supplementary concept word, unique identifier, synonyms]
31. 28 or 29 or 30
32. **27 and 31**

## Abbreviations

GRADE: Grading of Recommendations Assessment, Development and Evaluation
ICH-GCP: International Council for Harmonisation guideline for Good Clinical Practice Good Clinical Practice
HbA1C: Glycated haemoglobin
MET: Metabolic Equivalent of Tasks
SD: Standard deviation
WHO: World Health Organization
